# Non-covalent interactions involving halogenated derivatives of capecitabine and thymidylate synthase: a computational approach

**DOI:** 10.1186/s40064-016-1844-y

**Published:** 2016-02-24

**Authors:** Adhip Rahman, Mohammad Mazharol Hoque, Mohammad A. K. Khan, Mohammed G. Sarwar, Mohammad A. Halim

**Affiliations:** Bangladesh Institute of Computational Chemistry and Biochemistry, 38 Green Road West, Dhaka, 1205 Bangladesh; Department of Chemistry, University of Dhaka, Dhaka, 1000 Bangladesh; Department of General Studies, Jubail University College, Jubail Industrial City, 31961 The Kingdom of Saudi Arabia; Department of Chemistry, The Scripps Research Institute, 10550 North Torrey Pines Road, MB26, La Jolla, CA 92037 USA; Institut Lumière Matière, Université Lyon 1 – CNRS, Université de Lyon, 69622 Villeurbanne Cedex, France

## Abstract

Capecitabine, a fluoropyrimidine prodrug, has been a frequently chosen ligand for the last one and half decades to inhibit thymidylate synthase (TYMS) for treatment of colorectal cancer. TYMS is a key enzyme for de novo synthesis of deoxythymidine monophosphate and subsequent synthesis of DNA. Recent years have also seen the trait of modifying ligands using halogens and trifluoromethyl (–CF_3_) group to ensure enhanced drug performance. In this study, in silico modification of capecitabine with Cl, Br, I atoms and –CF_3_ group has been performed. Density functional theory has been employed to optimize the drug molecules and elucidate their thermodynamic and electrical properties such as Gibbs free energy, enthalpy, electronic energy, dipole moment and frontier orbital features (HOMO–LUMO gap, hardness and softness). Flexible and rigid molecular docking have been implemented between drugs and the receptor TYMS. Both inter- and intra-molecular non-covalent interactions involving the amino acid residues of TYMS and the drug molecules are explored in details. The drugs were superimposed on the resolved crystal structure (at 1.9 Å) of ZD1694/dUMP/TYMS system to shed light on similarity of the binding of capecitabine, and its modifiers, to that of ZD1694. Together, these results may provide more insights prior to synthesizing halogen-directed derivatives of capecitabine for anticancer treatment.

**Electronic supplementary material:**

The online version of this article (doi:10.1186/s40064-016-1844-y) contains supplementary material, which is available to authorized users.

## Background

Thymidylate synthase (TYMS) is a homodimeric protein with identical subunits (molecular weight for each unit ~35 kDa) and one of the most conserved protein entities in nature (Chu et al. 1991; Phan et al. 2001). It has long been well-known as a drug target, notably of 5-fluoro uracil (5-FU) and the anti-folate raltitrexed, for treatments of colorectal cancer (Chu et al. 1991; Phan et al. 2001; Costi et al. 2005). Both subunits of TS contribute to each of the two active sites; however, it shows “half-of-the-site activity” i.e. conformational switching between active and inactive states, thus only one subunit being active at a time (Świniarska et al. 2010; Luo et al. 2011). It is a key enzyme in folate metabolic pathway—thereby an essential precursor for biosynthesis of DNA, RNA and protein (Arooj et al. 2013; Hardy et al. 1987). TYMS catalyses the de novo pathway for the production of deoxythymidine monophosphate (dTMP), one of the three nucleotides which form thymine (a nucleic acid in DNA) and dihydrofolate from deoxyuridine monophosphate (dUMP) and 5,10-methylenetetrahydrofolate (mTHF) (Chu et al. 1991; Peters et al. 2002; Salo-Ahen and Wade 2011). The process involves a cycle, where dietary folate is reduced to dihydrofolate and then to tetrahydrofolate (THF) by dihydrofolate reductase while D_2_NADPH supplying necessary hydrogen. Serine transhydroxymethylase converts THF to mTHF. Meanwhile, dUMP binds to a receptor site of TYMS—prompting a configurational change to create a binding site for mTHF. Transfer of a methyl group to the uridine ring results in the formation of dihydrofolate and dTMP (Danenberg and Danenberg 1978; Santi et al. 1974). Because of its pivotal role in synthesis of DNA, TYMS remains one of the important target proteins in cancer chemotherapy.

Capecitabine (*N*^4^-pentyloxycarbonyl-5-deoxy-5-fluorocytidine) is a fluoropyrimidine-based novel oral prodrug designed by Miwa et al. This drug received the approval by FDA (US Food and Drug Administration) in 1998 and has been in use as a ligand for TYMS inhibition. A prodrugis an inactive chemical derivative of an active drug molecule which, after being administered specifically into the target cells or tumors, is converted to the desired form and ensures improved bioavailability. When delivered orally, capecitabine is absorbed through the intestinal wall of cells and converted to 5′-deoxy-5-fluorouridinein a three-step sequential enzymatic pathway, which includes the last-step tumor selective reaction catalyzed by the tumor-associated angiogenic factor thymidine phosphorylase—eventually leading to transformation into 5-fluorouracil (5-FU). 5-FU has been known as the mainstream antifolate that inhibits TYMS. Entering the cell, 5-FU is converted to 5-fluorodeoxyuridine monophosphate (FdUMP) which, mutually with the methyl donor-reduced folate, forms a covalent stable ternary complex with TYMS, thus preventing thymidine synthesis in cells. In addition, its alteration into fluorouridine triphosphate (FUTP) and fluorodeoxyuridine triphosphate (FdUTP) disrupts the function of RNA and DNA in tumor cells respectively (Stella et al. 1985; Papamichael 2000; Etienne et al. 2004; Longley et al. 2003; Park et al. 2002; Miwa et al. 1998; Ishikawa et al. 1998). The drawback of direct administration of 5-FU due to primary and secondary resistance and its potential toxicity to normal, non-tumored cells, however, has been a matter of concern. 5-FU undergoes rapid metabolic change by dihydropyrimidine dehydrogenase (DPD) in the mucosa of the gastrointestinal tract and the liver—limiting its oral bioavailability. In addition, adverse side-effects during its application—diarrhea, nausea and cardiovascular complexities for instance, have been reported (Mader et al. 1998; Saif 2009; Abou and Fadl 2009). Contrariwise, capecitabine is well-tolerated and enhances drug concentration at the tumor site, avoids complication associated with venous access and reduces cytotoxicity. Infact, stage III trials for treatment of colorectal cancer using capecitabine have been undertaken and shown markedly superior results, compared to 5-FU, with improved safety profiles (Miwa et al. 1998; Scheithauer et al. 2003; Schmoll and Arnold 2006).

In governing the mechanisms responsible for diverse ligand–protein complex systems such as the one mentioned above, non-covalent intermolecular forces play a pivotal role. Elucidation and quantification of non-covalent forces are central to pharmaceutical drug-design and lead optimization. Important non-covalent interactions include hydrogen bonding, electrostatic interactions, stacking interactions (cation–pi, anion–pi or pi–pi), van der Walls forces, and hydrophobic interactions (Meyer et al. 2003; Cerný and Hobza 2007; Dougherty 2007; Wheeler and Bloom 2014; Wheeler 2011; Varma et al. 2010). Recent years have witnessed the importance of non-covalent interactions involving halogen atoms, otherwise termed as halogen bonding, due to their intriguing chemical features. Halogen bonds have been identified in many of biological low-mass compounds and in complexes of biomolecules with halogenated ligands. Halogen atoms can either function as electrophilic species or as nucleophilic Lewis bases and display anisotropic charge distribution along the region of a C–X bond with an equatorial distribution of negative and positive charges—thereby creating what is called a σ-hole. A σ-hole maintains a diminished electron density site and entices an electronegative site of another molecule to interact. Such cases occur, especially those with larger atomic radii (chlorine, bromine and iodine) (Lu et al. 2009, 2012; Sirimulla et al. 2013). On the other hand, drug candidates containing fluorine show increased metabolic stability and membrane permeation, hence use of them has become commonplace. The small-size and very high electronegativity of fluorine attribute to its eccentric behaviors, which in turn contribute to its versatile interactions in biomolecular receptor-ligand moieties (Hagmann 2008; Smart 2001; Zhou et al. 2009).

This in silico study focuses on quantum mechanical and molecular docking analysis of thymidylate synthase (TYMS) against capecitabine and its halogenated derivatives, modified by trifluoromethane (–CF_3_), Cl, Br and I, to investigate compelling non-covalent interactions in order to attain a deeper understanding and interpret the core characteristics of such receptor-ligand synergy. In-silico approach, computer-aided drug design (CADD) in other words, has brought about a major revolution to facilitate the design and discovery of novel therapeutic solutions (Kapetanovic 2008). CADD has been utilized in diverse ways—choosing the most exact ligands and their target proteins from digital repositories such as DrugBank (Wishart et al. 2006), PubChem (Wang et al. 2009) or PDB (Berman et al. 2002), using softwares adept in sophisticated quantum mechanical calculations and 3-D computer graphics to model and/or manipulate potential inhibitors, carrying out molecular docking in order to foresight energetically favorable binding sites and interactions among the lead-candidates and target molecules with acute physicochemical and pharmaceutical details (Koutsoukas et al. 2011; Kapetanovic 2008; Tang et al. 2006; Song et al. 2009; Joseph-Mccarthy 1999). Discovery and marketing of a drug on average require around 7–12 years of research and cost from US$800 million to US $1.2 billion; however, CADD has been successful in minimizing the lengthy trial-and-error research cycle and gigantic costs involving drug discovery (Kapetanovic 2008; Tang et al. 2006). In this work, thermo-chemical and molecular orbital calculation for the drug candidates were performed. Each of the ligand molecules was subjected to flexible and rigid docking to elucidate binding energies, binding sites and characteristics of various non-bonding interactions among those complex receptor–ligand environments. In addition, the results have been compared to the previously determined crystal structure of ZD1694–dUMP–TYMS complex at 1.9 A resolution to identify the degree of superposition of the presently observed systems to that of a resolved one (Phan et al. 2001).

## Computational methods

### Optimization of drugs using quantum mechanical calculations

**Fig. 1:**
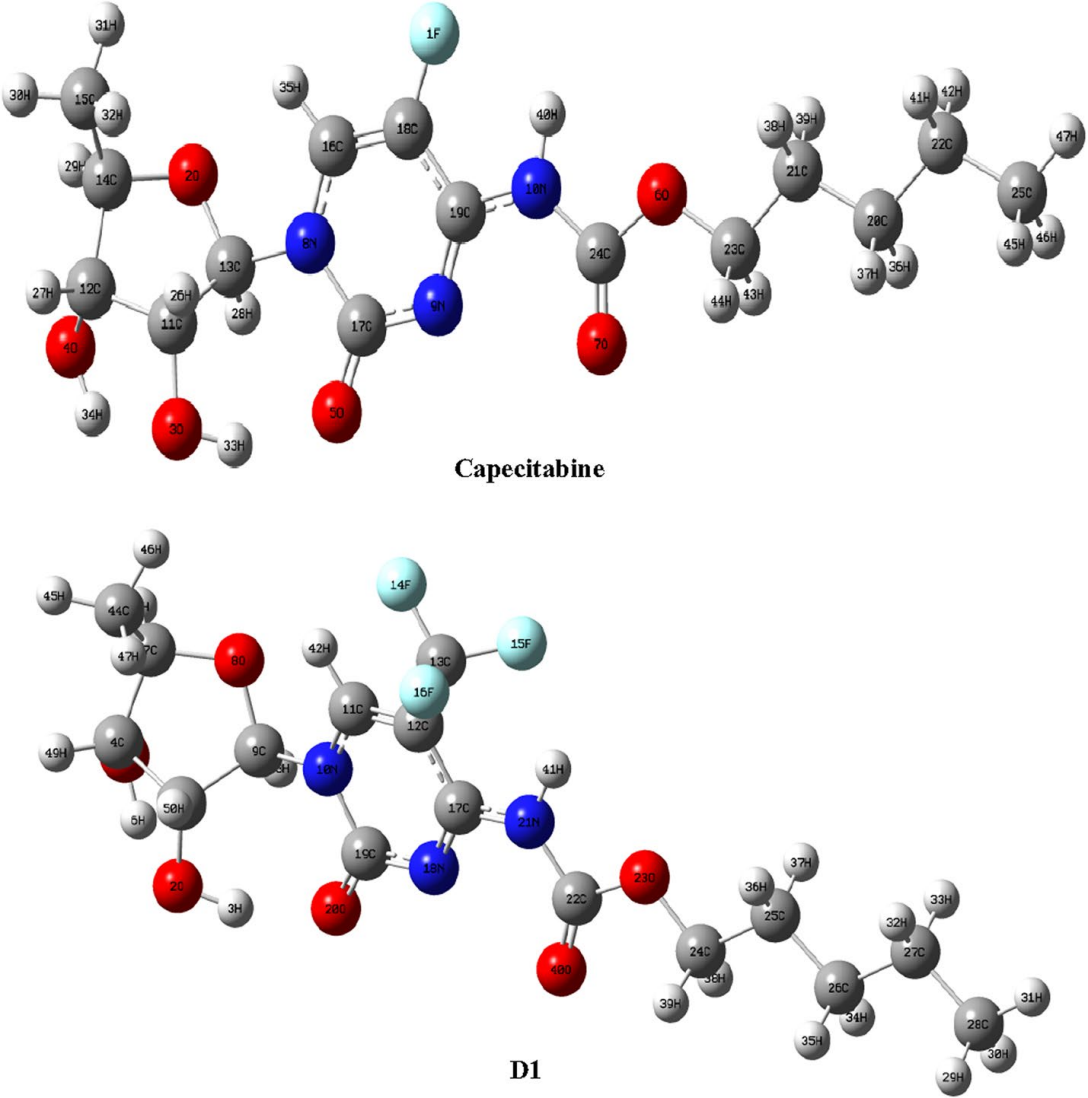
Optimized structure of capecitabine and its halogenated derivatives (**D1**) generated at B3LYP/MidiX level of theory

**Fig. 2:**
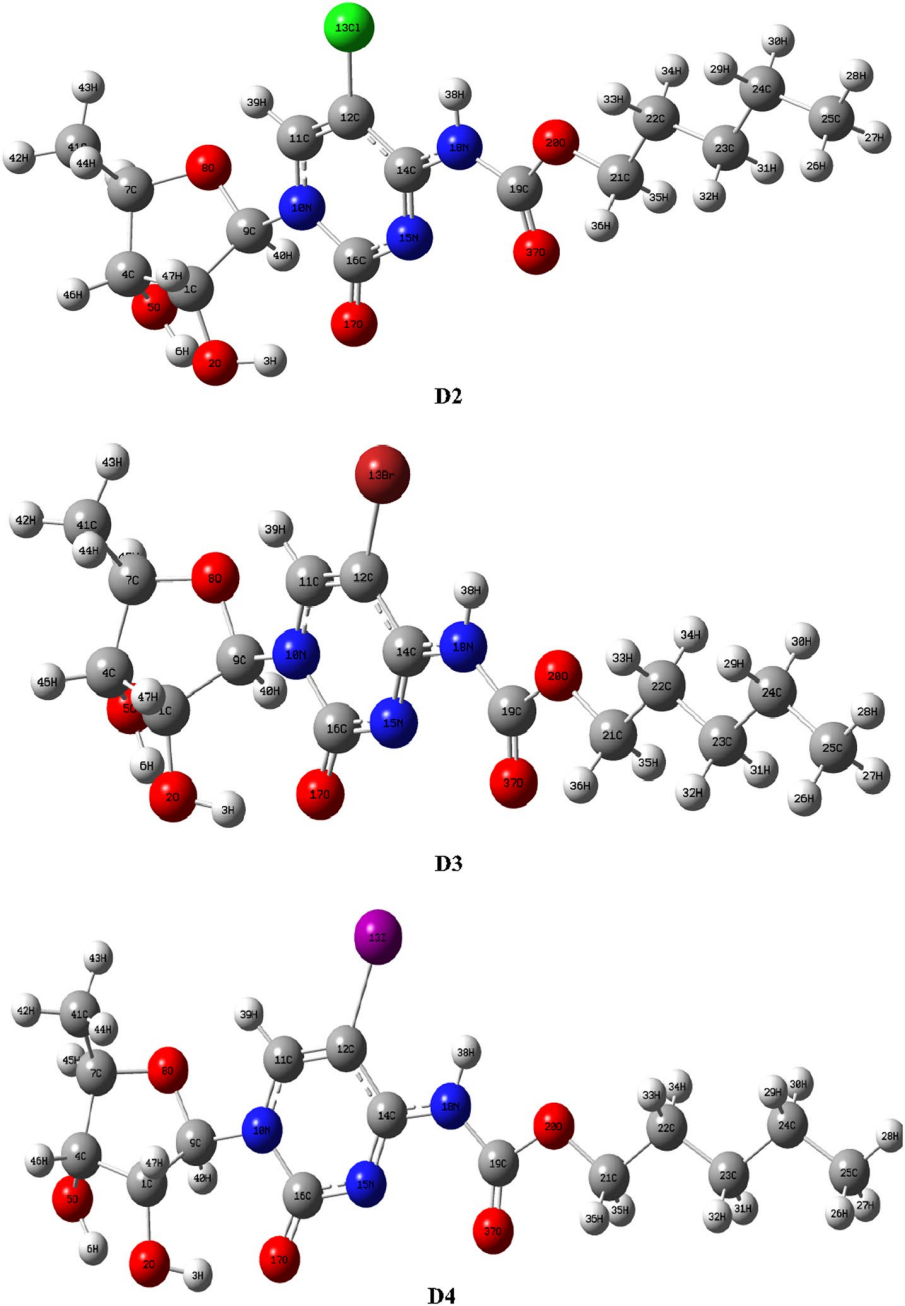
Optimized structure of halogenated derivatives (**D2**, **D3** and **D4**) generated at B3LYP/MidiX level of theory

Quantum mechanical calculations were performed using Gaussian 09 program-suit (Gaussian 09 Revision 2009). The structures of capecitabine was fully optimized utilizing density functional theory employing B3LYP/MidiX level of theory. The basis set is initially developed from the Huzinaga MIDI basis and more compatible for molecules with halogen atoms (Easton et al. 1996). The initial structure of capecitabine was manipulated replacing fluorine with –CF_3_, Cl, Br and I; the modified compounds, labeled from **D1**–**D4** sequentially (Figs. 1, 2), were then optimized using the same level of theory. Internal electronic energy, enthalpy, Gibbs free energy and dipole moment were investigated for each of the molecules. Energetics data of a system exploiting Gaussian 09 are produced by solving traditional thermodynamic and quantum mechanical equations. Contributions from each of translational, rotational, vibrational and electronic motions are considered to estimate the thermodynamic parameters of a system. Sum of electronic and thermal energies in a molecule is represented by the following equation (Gaussian 09 Revision 2009)

1 $${\text{Sum}}\;{\text{of}}\;{\text{electronic}}\;{\text{and}}\;{\text{thermal}}\;{\text{energies}} = \varepsilon_{0} + E_{\text{tot}}$$

where ε_o_ is the total electronic energy for a given system and *E*_tot_ is the correction to internal thermal energy when *E*_tot_ = *E*_t_ + *E*_r_ + *E*_v_ + *E*_e_. Similarly,

2 $${\text{Sum}}\;{\text{of}}\;{\text{electronic}}\;{\text{andthermal}}\;{\text{enthalpies}} = \varepsilon_{0} + H_{\text{corr}}$$

3 $${\text{Sum}}\;{\text{of}}\;{\text{electronic}}\;{\text{and}}\;{\text{thermal}}\;{\text{free}}\;{\text{energies}} = \varepsilon_{0} + G_{\text{corr}}$$

where *H*_corr_ and *G*_corr_ are the thermal corrections for enthalpy and Gibbs free energy for the given system respectively.

Frontier molecular orbital calculations were also conducted using the same level of theory. Considering the correlation of ionization potential (I) with HOMO and electron affinity (E) with LUMO according to Koopmans theorem, hardness (η) and softness (S) of the drugs were calculated according to the following equations (Pearson 1986)

$$\eta = \frac{{\left[ {\varepsilon {\text{LUMO}} - \varepsilon {\text{HOMO}}} \right]}}{2}$$

$${\text{S}} = \frac{1}{\eta }$$

### Preparation of protein

**Fig. 3:**
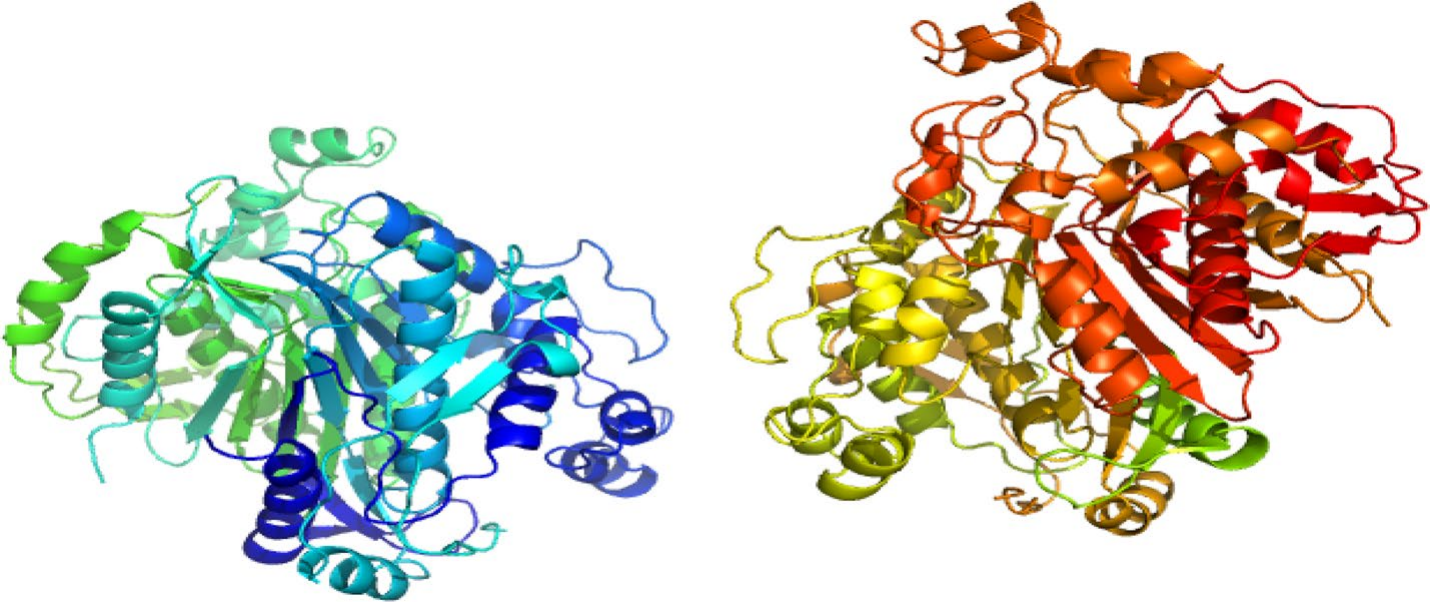
Crystal structure of thymidylate synthase (TYMS)

Crystal structure of thymidylate synthase (TYMS) was collected from Protein Data Bank (PDB, ID: 1HVY; Chain A) (Fig. 3). Prior to docking, heteroatoms, lipids and water molecules were removed from the crystal structure using PyMol (version 1.3) (DeLano 2002). Geometry and energy minimization of the crystal structure were carried out with Swiss-PDBViewer (version 4.1.0) employing GROMOS96 force field (Guex and Peitsch 1997; Scott et al. 1999). The ligand and protein structures were saved as *.pdb* files.

### Binding site and docking

The active binding pocket of TYMS was predicted using CASTp (Dundas et al. 2006), which found the largest pocket area and volume to be 1347.7 Å^2^ and 1976.3 Å^3^ respectively (Additional file 1: Figure S1 provides with some of the most favorable pocket area and volume alongside the amino acid residue chains. The colored region in the protein structure represented largest pocket area and volume). These information were used to generate the grid boxes during molecular docking.

Molecular docking is a method that predicts preferred orientation of a compound bound to a second during the formation of a stable complex and finds its frequent application in in silico pharmaceutical design. Current methods of docking include pose prediction, using docking algorithms and energy-based scoring functions that identify the optimal binding modes of a drug, i.e. energetically most favorable conformations, to the active sites of a target protein. Lower free energy means better ligand–protein binding (Thomsen and Christensen 2006; Gschwend et al. 1996; Kroemer 2007). Two types of docking algorithms are most common: flexible and rigid. In flexible docking, either one or both of the molecules involved in binding are considered as flexible objects, whereas rigid docking imposes conformational restriction on both the ligand and protein, thereby considering them as rigid solid objects (Zhou et al. 2007; Halperin et al. 2002). In the current work, both flexible and rigid docking for each of the ligand–protein entities were performed using Autodock Vina (Trott and Olson 2010). TORSDOF was set for all the ligands followed by the conversion of all rotatable bonds into non-rotatable during rigid docking. While performing flexible docking, ligand molecules were kept flexible and the protein was kept rigid. The grid boxes were constructed in such a manner that it covered the colored volumes corresponding to the binding pockets identified in Additional file 1: Figure S1. For both flexible and rigid docking, the dimensions of grid boxes were chosen as follows: 54.6895, 43.2718 and 54.3972 Å towards X, Y and Z co-ordinates respectively. Detailed analysis of the residues involved in non-covalent interactions between the ligands and protein was explored using Accelyrs Discovery Studio 4.1 (2013) and LigPlot+ (version 1.4.5) (Laskowski and Swindells 2011).

### Pharmacokinetic parameters

AdmetSAR online database has been utilized to generate the pharmacokinetic parameters related to drug absorption, metabolism and toxicity for the parent drug and its modifiers (Cheng et al. 2012). SDF (Structure Data File) and SMILES (simplified molecular-input line-entry system) strings were utilized throughout the generation process.

## Results and discussion

### Electronic and thermodynamic behavior of capecitabine and its derivatives

The current work has employed the equations mentioned in “Optimization of drugs using quantum mechanical calculations” section to produce thermochemical data that predict the energetic availability and flexibility of capecitabine and its halogenated derivatives, as depicted in Table 1. Exchange of F in the initial structure with bioisosteres –CF_3_, Cl, Br and I saw a gradual increase in the negative value of electronic and thermal energies, enthalpy and free energy, hence suggesting energetically and configurationally more preferable trifluoromethylated, chlorinated, brominated and iodinated molecules. To be specific, Br and I-substituted capecitabine molecules (**D3** and **D4**) showed marked changes in the thermochemical data, in contrast to that of –CF_3_ and Cl-substituted ones (**D1** and **D2**), suggesting greater atomic radii—halogen substituents allowing the modified drugs become more stable.

**Table 1:** Stoichiometry, electronic energy, enthalpy, Gibbs free energy (in Hartree) and dipole moment (in Debye) of Capecitabine and its halogenated derivatives

Ligands	Stoichiometry	Sum of electronic and thermal energies	Sum of electronic and thermal enthalpies	Sum of electronic and thermal free energies	Dipole moment
Capecitabine	C_15_H_22_FN_3_O_6_	−1299.926	−1299.925	−1300.009	6.6677
**D**_**1**_	C_16_H_22_F_3_N_3_O_6_	−1528.935	−1528.933	−1529.023	5.1524
**D**_**2**_	C_15_H_22_ClN_3_O_6_	−1651.218	−1651.217	−1651.301	5.3633
**D**_**3**_	C_15_H_22_BrN_3_O_6_	−3755.199	−3755.198	−3755.283	6.1689
**D**_**4**_	C_15_H_22_IN_3_O_6_	−8083.324	−8083.323	−8083.410	6.1851

Dipole moment is a useful parameter in the study of drug-receptor systems and plays a significant role for the formation of hydrogen bond in biological systems (Lien et al. 1982). The numerical value of dipole moment was highest for capecitabine. As listed in Table 1, high electronegativity of fluorine contributed to the overall high molecular dipole moment in capecitabine—partial charge on fluorine being −0.298 a.u., whereas the three fluorine atoms in –CF_3_ modified molecule **D1** showed even more electronegativity, ranging from −0.305 to −0.310 a.u. but a relatively overall lower dipole moment value. Partial charge for iodine in the iodinated structure **D4** was found to be +0.148 a.u. and the value of molecular dipole moment seemed to be relatively higher, indicating relatively higher polarity for **D4(**the partial charge maps of capecitabine and its derivatives are provided from Additional file 1: Figure S2).

### Frontier molecular orbitals

Frontier orbitals (FO) are general terms used to denote both highest occupied molecular orbital (HOMO) and lowest unoccupied molecular orbital (LUMO). Frontier orbitals’ energy values are important to determine chemical reactivity and the extent to which a drug interacts with a particular receptor. Even, energy of HOMO along can be a pivotal determinant to find a relationship between a class of drug’s activity and their electronic configuration, as reported by Snyder et al. (Snyder and Merril 1965). The energy gap between HOMO and LUMO predicts of a molecule’s kinetic and chemical stability. Larger FO gap concords with high kinetic stability, but low chemical reactivity, as it is energetically unfavorable for an electron to have it elevated from a low-energy HOMO to a relatively high-lying LUMO (Aihara 1999; Hoque et al. 2013).

Pictorial views of HOMOs and LUMOs of capecitabine and its four derivatives are shown in Additional file 1: Table S1. Frontier orbital energy gap in capeciabine was found to be 0.1778 a.u.; values for the four derivatives ranged from 0.1770 to 0.1889 a.u. (Table 2). The iodinated derivative **D4** showed the lowest energy gap among the derivatives, 0.1770 a.u., which is even lower than the initial structure—hence enhanced softness, least hardness and high chemical reactivity. The –CF_3_ incorporated structure, on the other hand, revealed that it’s energy gap was the most unfavorable one in the group towards chemical reactivity.

**Table 2:** Energy of HOMOs, LUMOs (a.u.), orbital gap, hardness and softness of Capecitabine and its derivatives

Ligands	ε_HOMO_	ε_LUMO_	Orbital Gap	Hardness	Softness
Capecitabine	−0.2394	−0.0616	0.1778	0.0889	11.24
**D1**	−0.2447	−0.0558	0.1889	0.0944	10.59
**D2**	−0.2379	−0.0573	0.1801	0.0901	11.10
**D3**	−0.2327	−0.0543	0.1784	0.0892	11.21
**D4**	−0.2316	−0.0546	0.1770	0.0850	11.76

### Molecular docking: binding energy of the receptor-ligand systems

Table 3 lists the active site free energies of binding for each of the current receptor-ligand systems and shows a comparison between the binding energy values obtained via flexible and rigid docking for each of the entities. The interaction energy for each of the ligand atoms is calculated for all the possible binding sites—discredited through a grid box—of the receptor, resulting in multiple values of binding energies for each system. For instance, flexible and rigid docking for each of the capecitabine-TYMS entities produced total eighteen possible energy values. However, we have chosen the values corresponding to the second pose of the ligands as they tend to superimpose most appropriately on ZD1694—as we shall see in the latter sections. Binding energies for capecitabine-TYMS interactions of −7.1 and −8.8 Kcal mol^−1^ for flexible and rigid dockings respectively demonstrated significant deviation into energy values. The modified molecules followed the same trend as well. Flexible docking of **D1**–**D4** against TYMS showed binding energy values from −8.0 to −7.4 Kcal mol^−1^, when rigid docking showed values from −9.5 to −8.2 Kcal mol^−1^. The trifluoromethylated and chlorinated ligands possessed the two most negative energy values among the group, making them favorable modified derivatives than the other two.

**Table 3:** Free energy of binding values (Kcal mol^−1^) for ligand-TYMS systems obtained from flexible and rigid docking

Systems	Free energy of binding	
	Flexible docking	Rigid docking
Capecitabine-TYMS	−7.1	−8.8
**D1**-TYMS	−8.0	−9.5
**D2**-TYMS	−7.9	−9.0
**D3**-TYMS	−7.6	−8.2
**D4**-TYMS	−7.4	−8.5

### Non-covalent interactions within the receptor–ligand systems

**Fig. 4:**
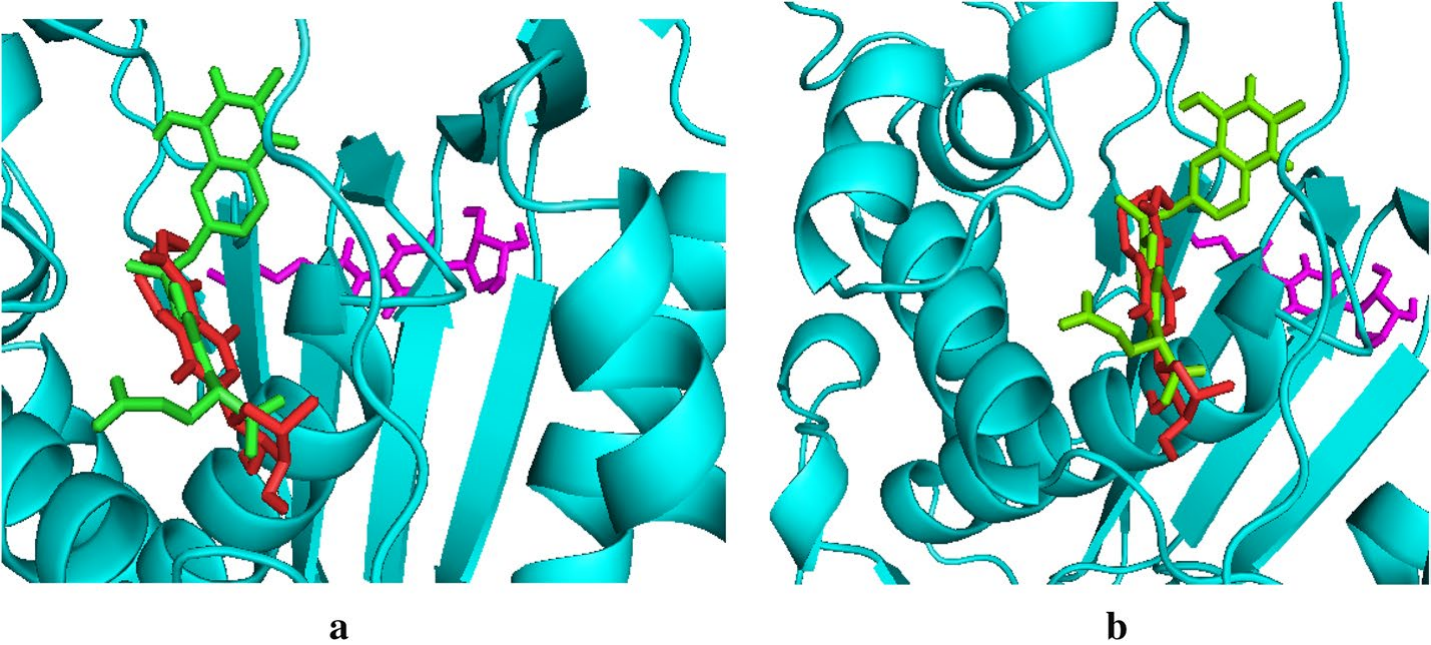
Superposition of **a** Capecitabine and **b** **D1** (trifluoromethylated ligand) on the ZD1694/dUMP/TYMS crystal structure resovlved at 1.9 Å. *Green*, *red* and *purple* molecules indicate ZD1694, flexibly docked and rigidly docked structures respectively

Crystal structure of ZD1694/dUMP/TYMS complex, in the earlier researches of Phan et al. confirmed the fact that a Cα–S and a O–H covalent bonding at Cys195 and His196 of hTYMS are involved during the transformation of dUMP to dTMP (Phan et al. 2001). ZD1694 in the complex acts as a competitive binder against antifolate THF and inhibits the transformation required for DNA synthesis. The local environment of ZD1694/dUMP/TYMS in their work showed a direct N–H–O contact between ZD1694 molecule and Asp218 as well as Gly222. The local environment, with all hydrophobic contacts being taken into consideration, has been regenerated for the purpose of this work—as depicted by Additional file 1: Figure S4. Possible non-covalent interactions analogous to the most negative free energies of binding in capecitabine-TYMS complex have also been shown in Table 4, a thorough checking of which elucidates that flexible docking provides with more interaction sites—compared to that of rigid docking. Moreover, further comparison between Capecitabine/dUMP/TYMS and ZD1694/dUMP/TYMS complexes shows the local environment to be nearly identical in case of flexibly docked molecules. For instance, non-bonding hydrophobic contacts surrounding ZD1694 in the regenerated structure involved the following amino acid residues—Lys77, Phe80, Gly87, Ile108, Gly222, Phe225, Tyr 258 and Met311. On the other hand, flexibly docked capecitabine showed hydrophobic contacts involving Arg78, Val79, Phe80, Ile108, Leu221, Gly222, Phe225 and Met309; interaction sites are found completely different, however, in the rigid-docked molecule. As Fig. 4 would suggest, ZD1694 and flexibly docked capecitabine are well superimposed on each other-taking into account that the geometry of the latter here resembles only one of the nine poses—when the rigidly docked drug is significantly away. The case is same for the modified derivatives where rigidly docked ligands are observed not to superimpose on the crystal structure of ZD1694. Some more evidences will be found in Additional file 1: Figure S5, where the non-bonding interactions involving rigid ligands have been shown. We, therefore, shall mostly focus on the non-bonding interactions involving flexibly docked ligands.

**Table 4:** Comparison between the non-covalent interactions between Capecitabine and TYMS from flexible and rigid docking

Systems	Non-covalent interactions					
	Flexible docking			Rigid docking		
	Contacts	Bonding type	Bond distance (Å)	Contacts	Bonding type	Bond distance (Å)
Capecitabine- TYMS	O–H–O Arg78	Hydrogen	2.31	O–H–N Val62	Hydrogen	3.05
	O–H–C Val79	Carbon	2.67	C–C–S Cys180	Alkyl	3.61
	O–H–N Phe80	Hydrogen	2.83	Alkyl–alkyl Leu198	Alkyl	3.69
	O–H–C Ile108	Carbon	2.80	F–C–O Gly211	Halogen	3.35
	Alkyl-π Ile108	Alkyl-π	5.45	F–O Leu212	Halogen	2.85
	Alkyl-π Leu221	Alkyl-π	4.47	F–H–C Tyr213	Carbon	2.84
	F–O Leu221	Halogen	3.26	C–H–O Leu252	Carbon	2.87
	F–H–C Gly222	Carbon	2.35			
	Alkyl-π Phe225	Alkyl-π	4.71			
	F–C–π Phe225	Alkyl-π	4.43			
	C–C–S Met309	Alkyl	4.88

The nature of the non-bonding interactions varied from conventional hydrogen bonding to stacking interactions, donor–donor and weak van der Walls interactions. For instance, in capecitabine/dUMP/TYMS, F–O and F–H–C halogen bonding at Leu221 and Gly222 occurred. Rigid docking, however, predicted such interaction at Leu212. Here it is worth mentioning that despite reports suggested that halogen atoms with partial positive charges take part in halogen bonding more frequently, electronegative fluorine atoms in these cases were observed to form such interaction (Politzer et al. 2007). Phe225 was found to donate its π-electrons cloud towards the alkyl chain and the carbon attached to F of the drug, thus forming multiple π-stacking interactions. Here the phenyl residue shifts away from the ligand compared to that of ZD1694-Phe225 interactions—as the distance increases to 4.71 from 4.50 Å. A sulphar interaction occurs at Met309 with a bond distance 4.88 Å. Rigid docking, however, shows no interaction at the peripherical amino acid residues to Met311. An alkyl–alkyl weak interaction, however, occurs at Leu198—which is close to the Cys195 and His196. As observed comparing the interaction listed in Table 4, key interactions between TYMS side chains and ZD1694 are mostly, if not entirely, preserved in the flexibly docked capecitabine. The preservation continues in the modified structures as well as we shall see in the upcoming discussions.

**Fig. 5:**
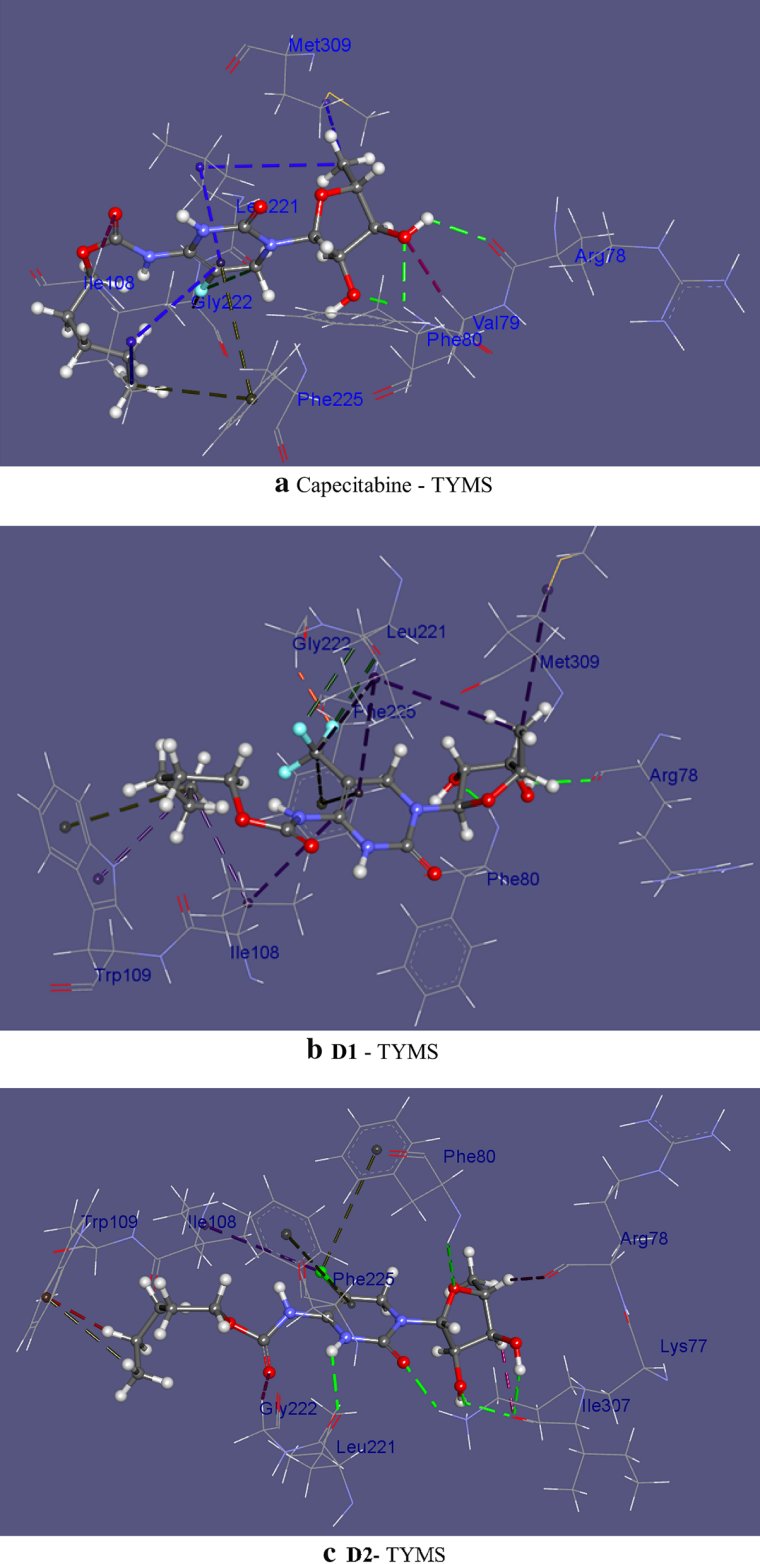
Non-covalent interactions among **a** Capecitabine-TYMS, **b** **D1** (trifluoromethylated)-TYMS and **c** **D2** (chlorinated)-TYMS generated by discovery studio

**Fig. 6:**
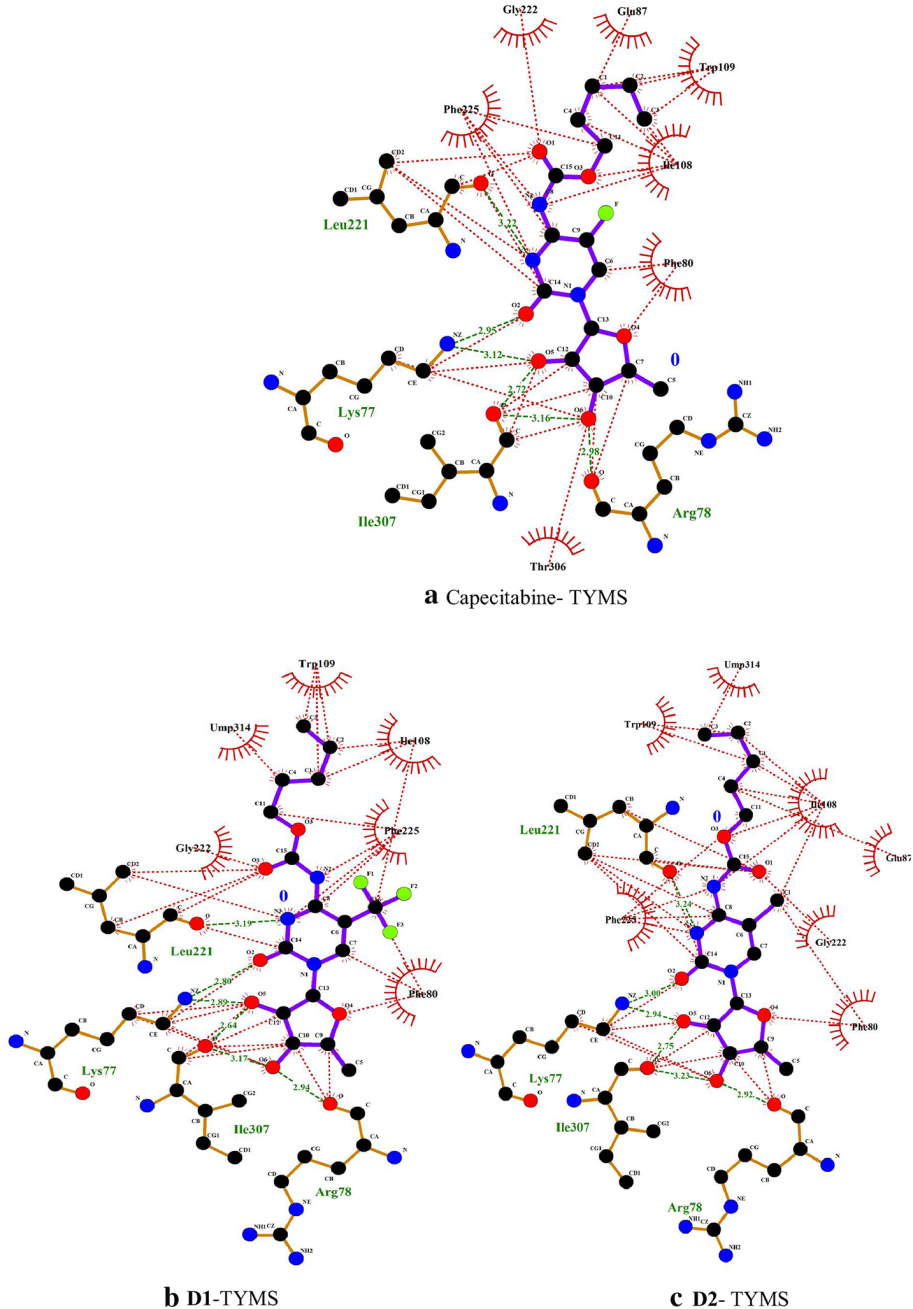
Non-bonding and hydrophobic binding sites (flexible docking) involving **a** Capecitabine, **b** –CF_3_ modified (**D1**) and **c** chlorinated derivative (**D2**) with TYMS

The value of trifluoromethyl (–CF_3_) group for its pharmacological activity has been acknowledged since the 50’s of last century. The widespread use in pharmaceutical products has been witnessed in recent years due to its unique chemical and physiological stability and the fact that –CF_3_ group is lipophilic in nature—an important determinant for increasing the solubility of drugs and letting it penetrate through cell membrane more easily (Yale 1959; Ji et al. 2011). Flexible docking showed that like capecitabine, a fluorine atom (F15) of the –CF_3_ group formed 2.82 Å long halogen bond with Leu221 and an F–C interaction with Gly222. Another F atom demonstated an anion-π stacking with the delocalized electron cloud of Phe225 (Fig. 5). A π-alkyl interaction at Trp109 was observed here—analogous to the interaction in ZD1694 complex. Lys77 was found to interact at the O of furan ring of the chlorinated derivative **D2**. Likewise, the halogen atom was found to form stacking and alkyl interactions with variable bond distances at Phe80 and Ile108. A π–π stacking was observed at Phe225 as well (Fig. 5). Nature of the residual environment surrounding the brominated ligand **D3** was merely identical to **D1** and **D2**—except Br forming an alkyl interaction at Ile108 as shown by the types of non-bonding interactions and the corresponding bond distances at (**D1**–**D4**)-TYMS in Table 5 (Additional file 1: Figure S3 illustrates the non-bonding scenario of **D3** and **D4** with TYMS). Figure 6 illustrates a more rigorous view of the interactions, including the hydrophic binding sites at the periphery, of capecitabine, **D1** and **D2** ligands, as these three molecules are closer with respect to the binding energy values to one another.

**Table 5:** Selected non-covalent interactions among drugs **D1**–**D4** and TYMS obtained via flexible docking

Systems	Contacts	Bond type	Bond **distances (Å)**	Systems	Contacts	Bond Type	Bond **distances (Å)**
**D1**-TYMS	O–H–O Arg78	Hydrogen	2.43	**D3**-TYMS	O–H–N Lys77	Hydrogen	2.44
	O–H–N Phe80	Hydrogen	2.00		O–O Arg78	Donor	3.00
	alkyl–alkyl Ile108	Alkyl	4.21.5.26		C–H–O Arg78	Carbon	2.43
	alkyl-π Trp109	Alkyl-π	4.20,4.29		O–H-N Phe80	Hydrogen	2.90
	F–O Leu221	Halogen	2.82		Br–alkyl Ile108	Alkyl	5.09
	alkyl–alkyl Leu221	Alkyl	4.61,5.32		Alkyl-π Trp109	Alkyl-π	3.83, 4.81
	F–H–C Gly222	Carbon	3.26		N–H–O Leu221	Hydrogen	2.67
	F–C–π Phe225	Alkyl-π	4.54		O–H–C Gly222	Carbon	2.58
	C–C–S Met309	Alkyl	4.79		π–π Phe225	π stack	5.41
					O–H–O Ile307	Hydrogen	2.53
					C–H–O Ile307	Carbon	2.79
**D2**-TYMS	O–H–N Lys77	Hydrogen	2.21	**D4**-TYMS	O–H–N Lys77	hydrogen	2.48
	O–O Arg78	Donor	2.92		C–H–O Arg78	Carbon	2.35
	C-H–O Arg78	Carbon	2.46		O–H–N Phe80	Hydrogen	2.93
	O–H–N Phe80	Hydrogen	2.80		I-alkyl Ile108	Alkyl	5.18
	C–H–π Phe80	Alkyl- π	5.05		C–H–π Trp109	Alkyl-π	2.42, 4.33
	Alkyl–alkyl Ile108	Alkyl	4.77		Alkyl–alkyl	Alkyl	5.09
	C–H–π Trp109	Alkyl- π	2.52, 4.35		Leu221	carbon	2.54
	Cl–O Leu221	Halogen	2.00		C–H–O Gly222	π stack	5.42
	O–H–C Gly222	Carbon	2.60		π–π Phe225	Hydrogen	2.18, 1.97
	π–π Phe225	π stack	5.16		O–H–O Ile307		
	O–H–O Ile307	Hydrogen	2.18, 2.34

In an article published in 2009, Mairal et al. investigated the potential role of iodine in inhibiting Transthyretin Fibrillogenesis in which they discussed on the binding pockets of Transthyretin (TTR) that could accommodate iodine; using Diflusinal and its derivatives as model compounds, they showed that iodine-inserted binding compounds could become crucial in inhibiting TTR activities for treatment of TTR-related amyloidosis (Mairal et al. 2009). Some other recent researches have also focused on pharmaceutical utilities of iodinated drugs (Barattin et al. 2010; Bois et al. 1998). The thermodynamic, frontier orbital and binding affinity data suggested good energetic availability of compound **D4**. Same as the **D3** derivative, flexible docking produced halogen-alkyl interactions with Ile108 (bond-length 5.18 Å). Partial positive charge of iodine, evidenced by partial charge map of **D4** (Additional file 1: Figure S2), might have been a factor behind such interactions. As Table 5 suggests, D4 shows similar types of interactions with the same amino acids compared to its counterparts, with the bond types varying among hydrogen bonding, alkyl interactions and π-stacking.

### Pharmacokinetic properties of the drugs

Inhibition constant K_i_ of all the drugs have been calculated using the equilibrium E + I ↔ EI, where E is the enzyme and I is the inhibitor molecule (the reference concentrations for all the entities have been considered 1 mol L^−1^ for the calculations) and the relationship

$$\ln {\text{K}}_{\text{b}} = - \ln {\text{K}}_{\text{i}} ,$$

where $\ln {\text{K}}_{\text{b}} = - \Delta {\text{G}}/{\text{RT}}, \, \Delta {\text{G}} = {\text{free}}\;{\text{energy}}\;{\text{of}}\;{\text{binding}}$ and presented in the last row of Table 6. All of the drugs are non-carcinogenic, according to the ADME (absorption, distribution, metabolism, and excretion) analysis and possess a class III acute oral toxicity. The LD50 values also support the level of the acute toxicity, as they reveal good amount of tolerance against oral toxicity. This means that the drugs are relatively safer for oral delivery, and the –CF_3_ modified drug is the safest in the group—as seen by the LD50 values depicted in Table 6. The drugs are supposed to be absorbed without much complexity as the human intestinal absorption was found positive for all the ligands (Shen et al. 2010). All the drugs are P-glycoprotein non-inhibitor, when the probability is higher for the parent and –CF_3_ modified ligands. Inhibition of P-glycoprotein affects negatively a drug’s bioavailability and the extent of drug metabolism and intestinal absorption (Broccatelli et al. 2011). The drugs do, however, show a positivity considering blood brain barrier, predicting that the drugs will go through the BBB. Adverse drug–drug interactions and severe cardiac side effects can be avoided with capecitabine and the modified molecules, as the molecules are both CyP450 2C9 and hERG non-inhibitor (Shen et al. 2010; Wang et al. 2012; Cheng et al. 2011).

**Table 6:** Selected pharmacokinetic parameters of capecitabine and its derivatives

Parameters	Capecitabine	**D1**	**D2**	**D3**	**D4**
Blood brain barrier	+ (0.6064)	+ (0.5585)	+ (0.5248)	+ (0.5162)	+ (0.5000)
Human intestinal absorption	+ (0.9513)	+ (0.9569)	+ (0.9524)	+ (0.9322)	+ (0.7879)
P-glycoprotein inhibitor	Non-inhibitor (0.7514)	Non-inhibitor (0.6987)	Non-inhibitor (0.6305)	Non-inhibitor (0.5777)	Non-inhibitor (0.6890)
CYP450 2C9 Inhibitor	Non-inhibitor (0.7673)	Non-inhibitor (0.7421)	Non-inhibitor (0.7490)	Non-inhibitor (0.7585)	Non-inhibitor (0.7696)
Human ether-a-go–go-related (hERG) gene inhibition	Non-inhibitor (0.7124)	Non-inhibitor (0.6884)	Non-inhibitor (0.7261)	Non-inhibitor (0.7313)	Non-inhibitor (0.7409)
Acute oral toxicity	III	III	III	III	III
Rat acute toxicity, LD50 (mol/kg)	2.4690	2.4907	2.4453	2.4229	2.4365
Ki at 298 K (nM)	3116.86	635.71	753.50	1242.24	1834.69

Probability values related to each of the parameters are given in the parenthesis

## Conclusion

Halogenation in capecitabine contributed to changes in its physicochemical properties. Thermodynamic calculation demonstrated the stability of capecitabine and its modified derivatives (**D1**–**D4**). Free energy and entropy estimation revealed relatively higher negative values, particularly for brominated and iodinated moieties (**D3** and **D4**). The drug molecules were polar in nature confirmed by calculation of dipole moment. Frontier orbital calculations revealed increased hardness for the –CF_3_ modified drug **D1** and increased softness for the iodinated drug **D4**; moreover, softness values of the drugs, except fluorine, followed an increasing trend with the substitution of halogens of greater atomic radii. The –CF_3_ modified ligand was found to possess the most negative binding energy value. Molecular docking, both flexible and rigid, exposed the free energies of binding for each ligand–TYMS interaction, when in each case the value obtained by rigid docking was found slightly more negative than that obtained by flexible docking. However, the flexibly docked ligands could be superimposed better on ZD1694 within the crystal structure of TYMS, when rigidly docked molecules showed inconsistency in binding to the proper active sites. Docking identified wide-range non-bonding interactions among the atoms of the drugs and amino acid units of TYMS. Flexible docking, for each ligand–receptor systems, revealed significant halogen interactions as well. Such compilation of information may prove worthwhile for more cost-effective and precise drug-designing and/or manipulation for TYMS inhibition.

## Additional file

10.1186/s40064-016-1844-y **Figure S1** to **Figure S5** depict binding pockets, partial charge maps and non-covalent interactions. **Table S1** shows the Frontier orbitals.
