# Supplementary material for: Non-covalent interactions involving halogenated derivatives of capecitabine and thymidylate synthase: a computational approach
Source: Springerplus. 2016 Feb 24;5:146. doi: 10.1186/s40064-016-1844-y (PMC4764604; doi:10.1186/s40064-016-1844-y)
Supplement: Supplementary file 1 — 10.1186/s40064-016-1844-y Figure S1 to Figure S5 depict binding pockets, partial charge maps and non-covalent interactions. Table S1 shows the Frontier orbitals. [file 40064_2016_1844_MOESM1_ESM.docx]

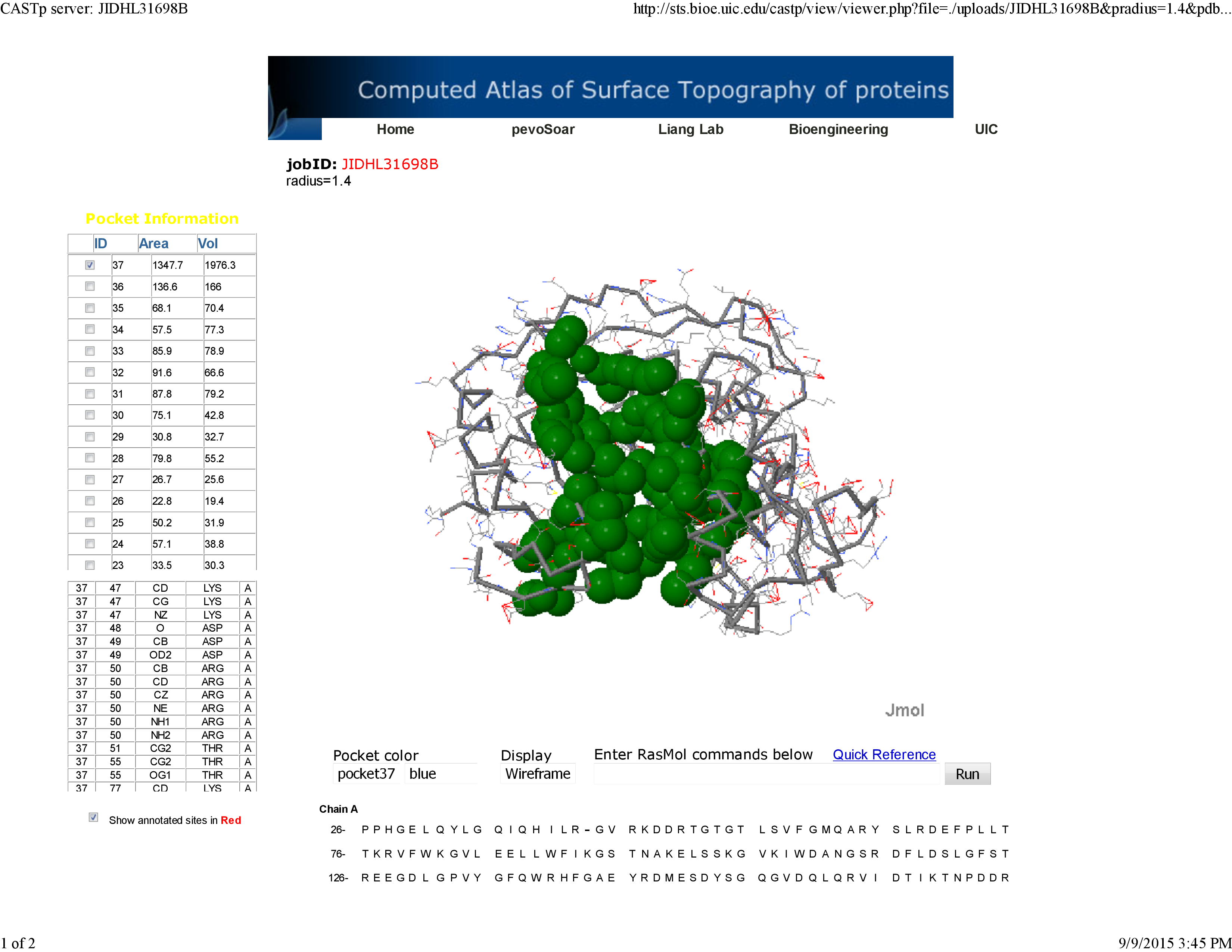


(a)

|  |
| --- |

(a)

**Capecitabine**

(a)

| **Chain A** |  |  |  |  |  |  |  |  |  |  |  |  |  |  |  |  |  |  |  |  |  |  |  |  |  |  |  |  |  |  |  |  |  |  |  |  |  |  |  |  |  |  |  |  |  |  |  |  |  |  |  |  |  |  |
| --- | --- | --- | --- | --- | --- | --- | --- | --- | --- | --- | --- | --- | --- | --- | --- | --- | --- | --- | --- | --- | --- | --- | --- | --- | --- | --- | --- | --- | --- | --- | --- | --- | --- | --- | --- | --- | --- | --- | --- | --- | --- | --- | --- | --- | --- | --- | --- | --- | --- | --- | --- | --- | --- | --- |
| 26- | P | P | H | G | E | L | Q | Y | L | G |  | Q | I | Q | H | I | L | R | - | G | V |  | R | K | D | D | R | T | G | T | G | T |  | L | S | V | F | G | M | Q | A | R | Y |  | S | L | R | D | E | F | P | L | L | T |
| 76- | T | K | R | V | F | W | K | G | V | L |  | E | E | L | L | W | F | I | K | G | S |  | T | N | A | K | E | L | S | S | K | G |  | V | K | I | W | D | A | N | G | S | R |  | D | F | L | D | S | L | G | F | S | T |
| 126- | R | E | E | G | D | L | G | P | V | Y |  | G | F | Q | W | R | H | F | G | A | E |  | Y | R | D | M | E | S | D | Y | S | G |  | Q | G | V | D | Q | L | Q | R | V | I |  | D | T | I | K | T | N | P | D | D | R |
| 176- | R | I | I | M | C | A | W | N | P | R |  | D | L | P | L | M | A | L | P | P | C |  | H | A | L | C | Q | F | Y | V | V | N |  | S | E | L | S | C | Q | L | Y | Q | R |  | S | G | D | M | G | L | G | V | P | F |
| 226- | N | I | A | S | Y | A | L | L | T | Y |  | M | I | A | H | I | T | G | L | K | P |  | G | D | F | I | H | T | L | G | D | A |  | H | I | Y | L | N | H | I | E | P | L |  | K | I | Q | L | Q | R | E | P | R | P |
| 276- | F | P | K | L | R | I | L | R | K | V |  | E | K | I | - | D | F | K | A | E | D |  | F | Q | I | E | G | Y | N | P | H | P |  | T | I | K | M | E | M | A | V |  |  |  |  |  |  |  |  |  |  |  |  |  |

(b)

Figure S1: (a) Binding Pocket of TYMS- chain A (represted by green colored region) and (b) Amino-acid residues in chain A of TYMS


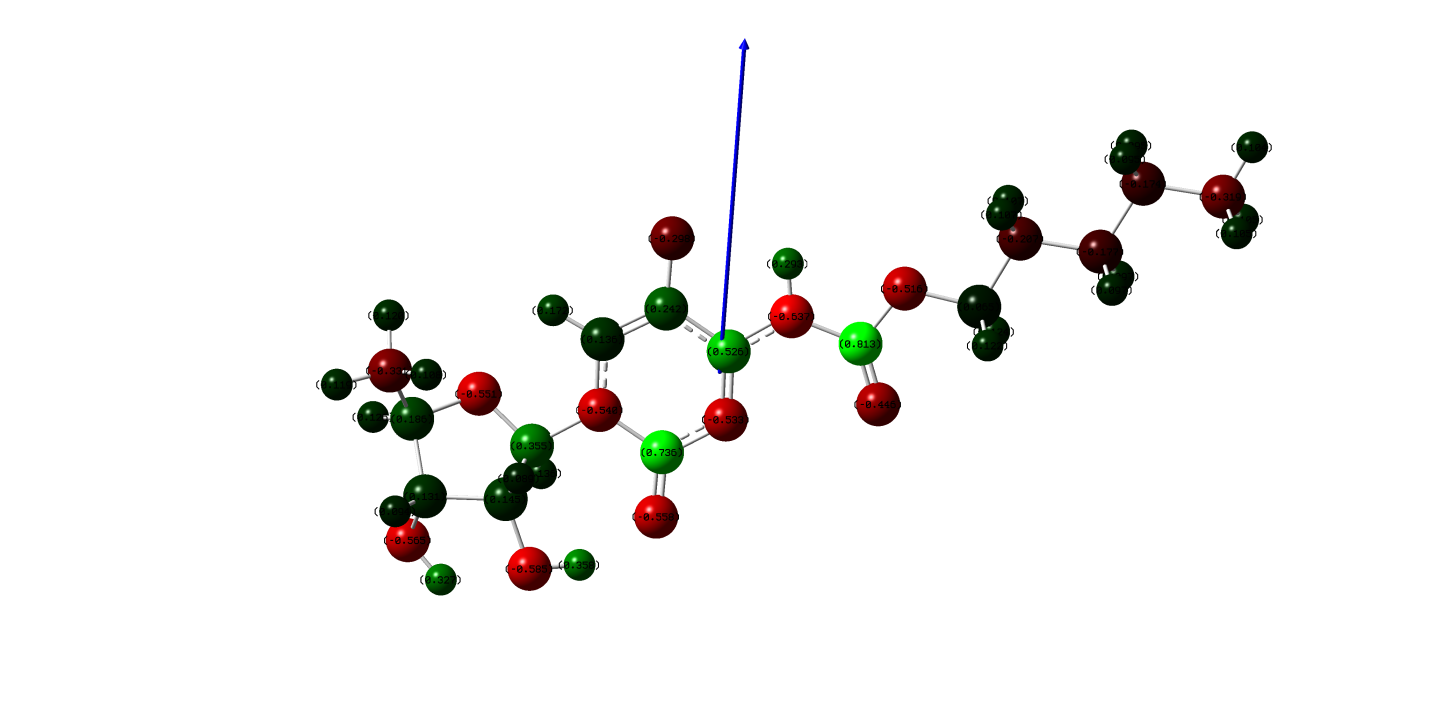


Capecitabine


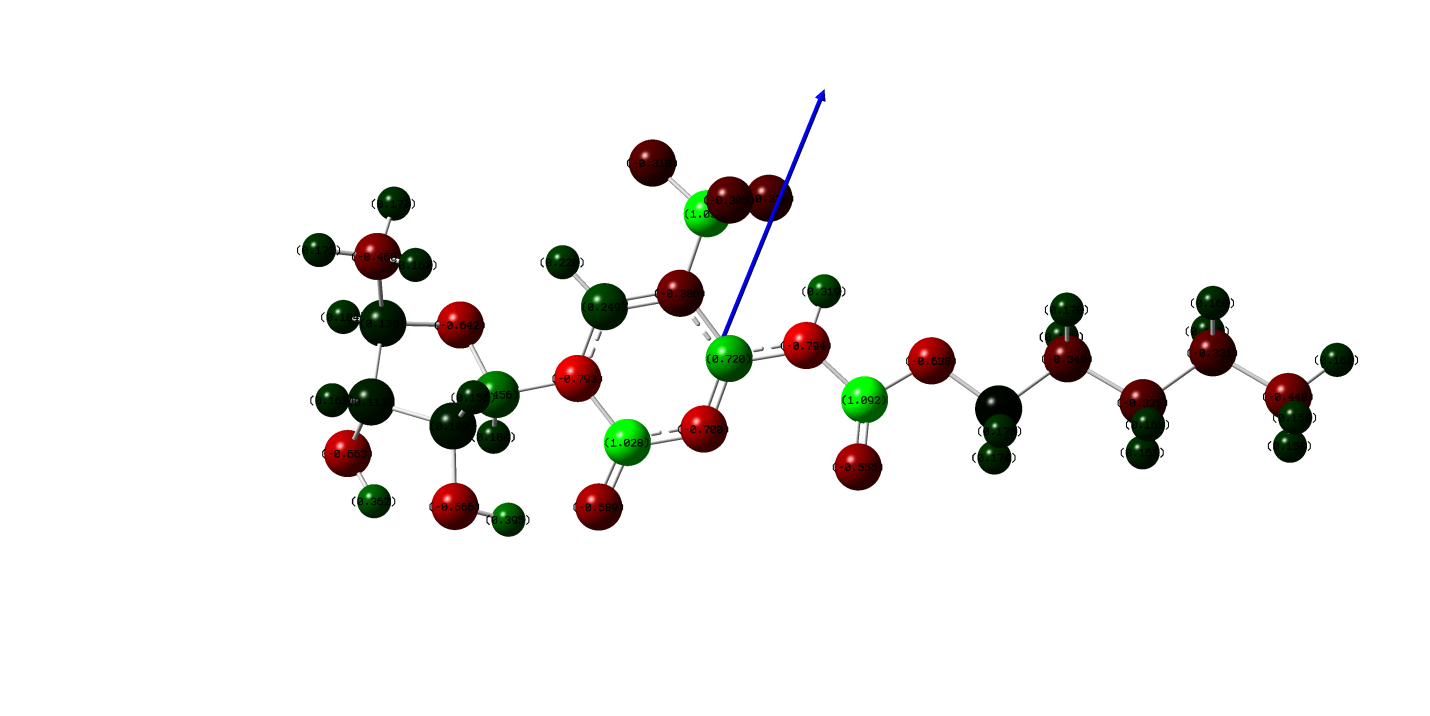


**D1**


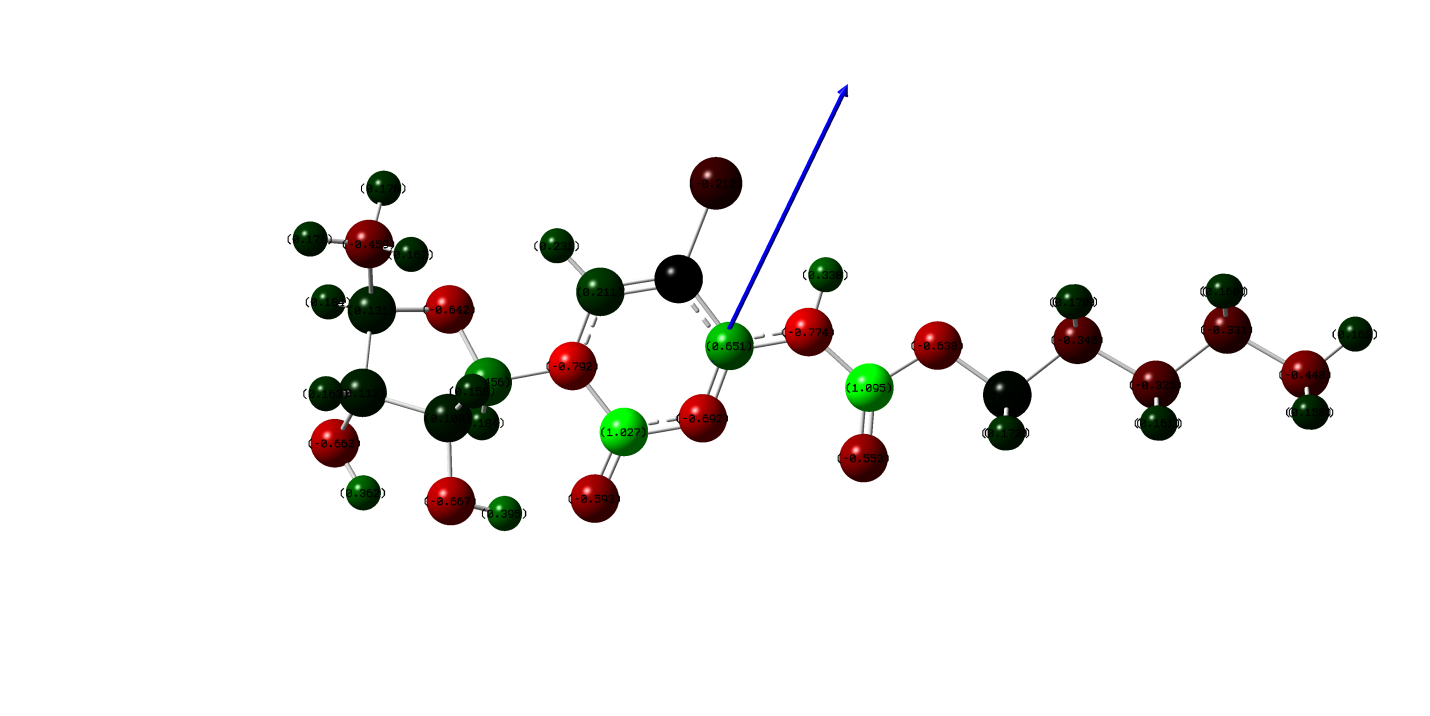


**D2**


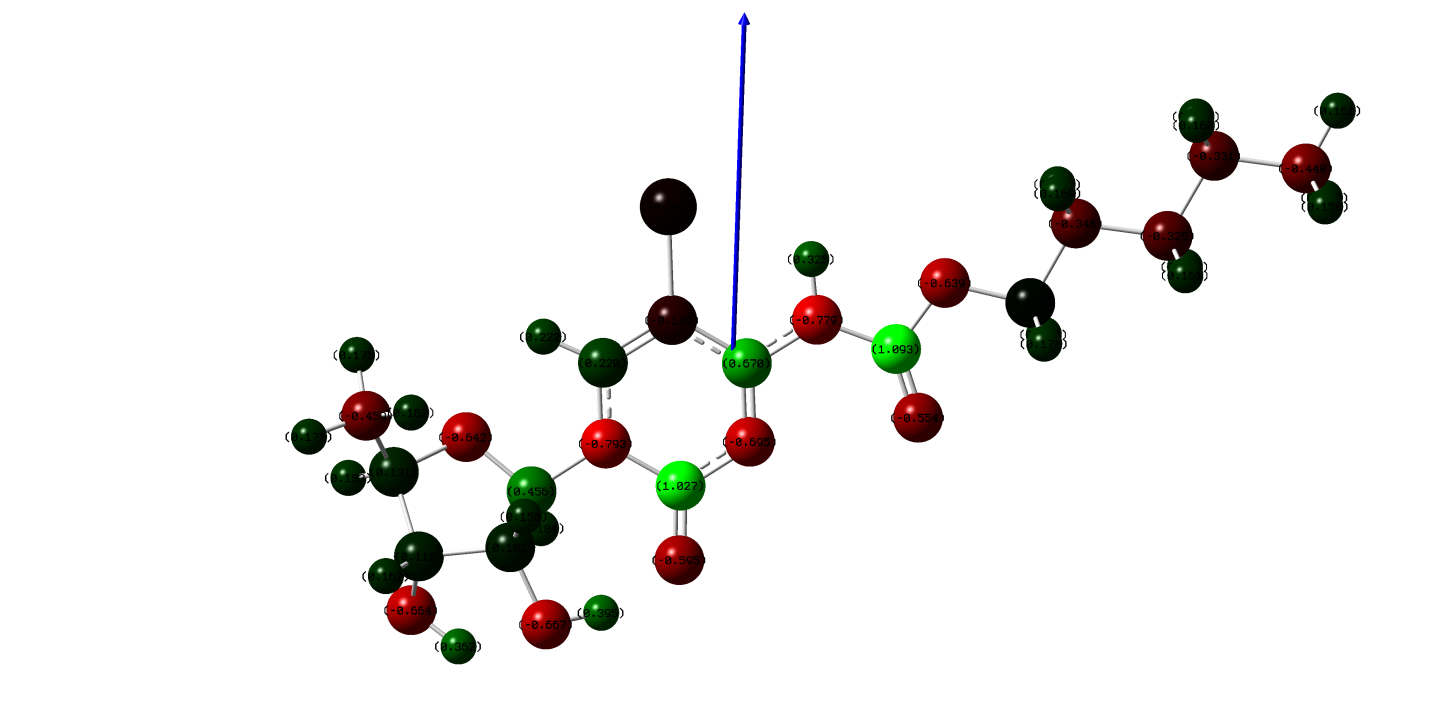


**D3**


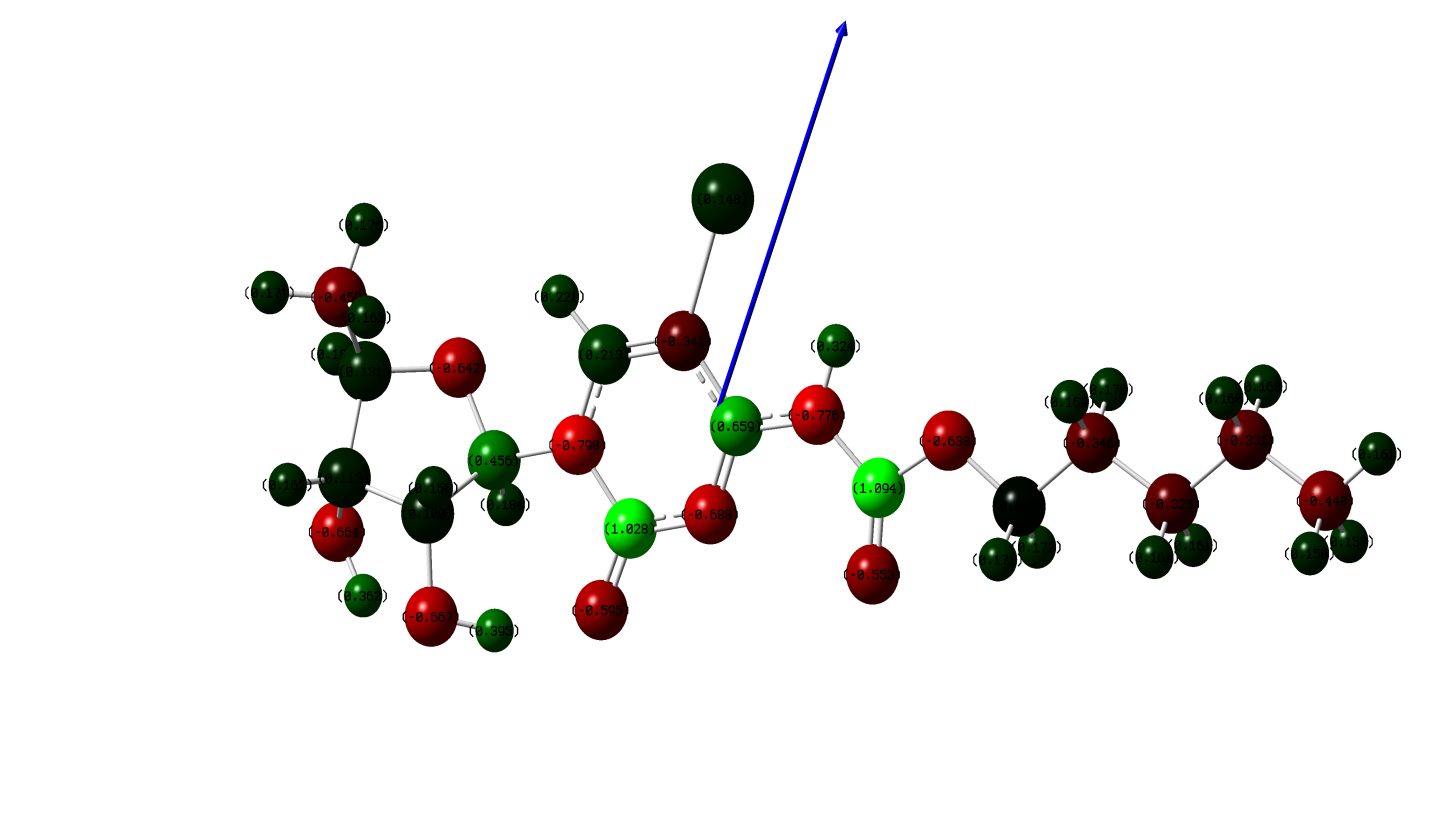


**Figure S2** : Partial charge maps of capecitabine and its halogenated derivatives (**D1**-**D4**)

**D4**

**Table S1** : Frontier molecular orbitals (HOMOs and LUMOs) of capecitabine and its halogenated derivatives

| Ligands | HOMO | LUMO |
| --- | --- | --- |
| Capecitabine | 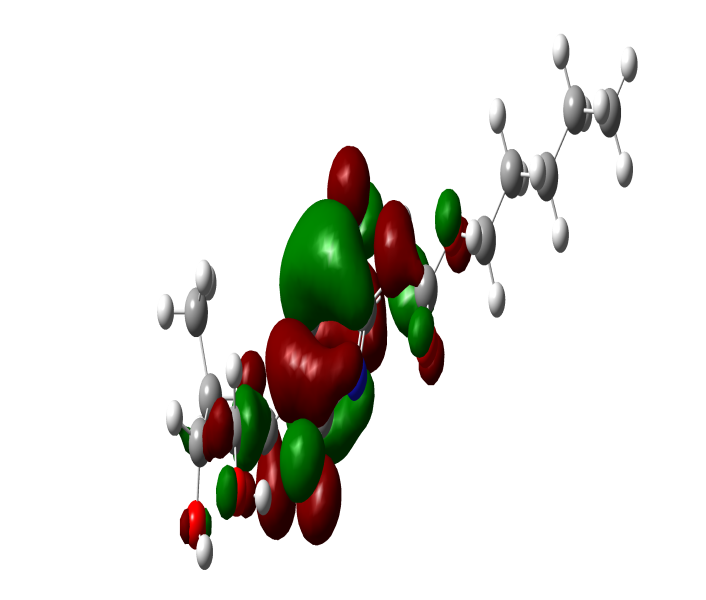 | 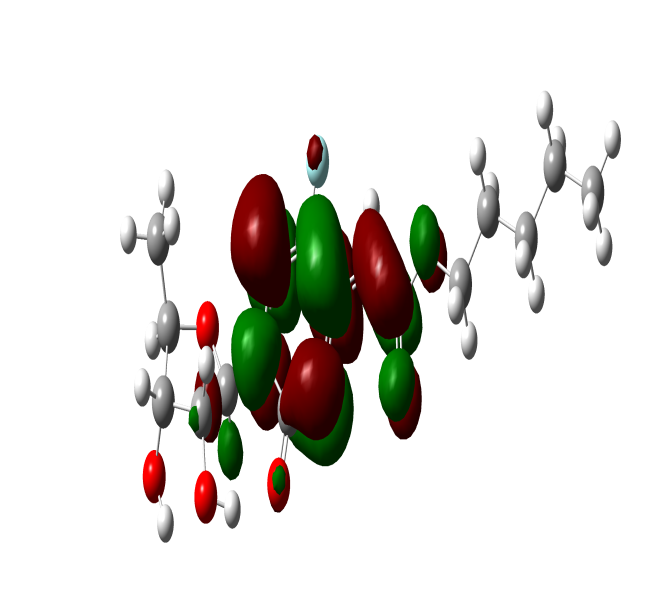 |
| D1 | 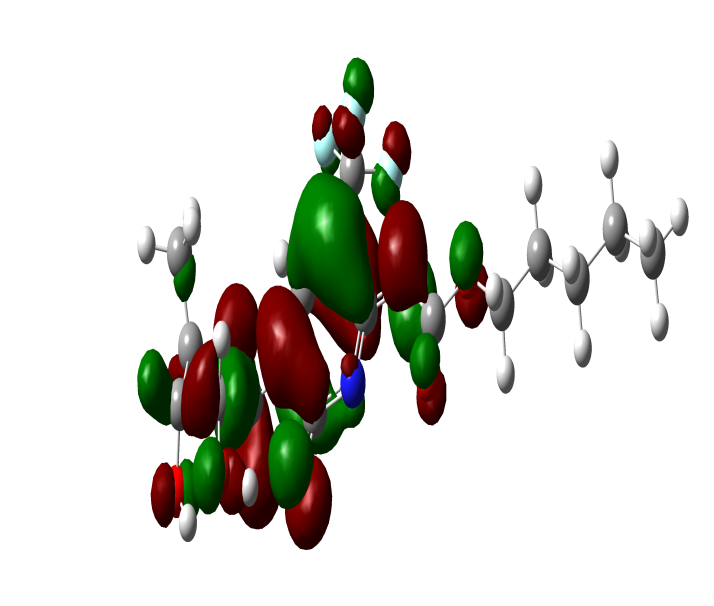 | 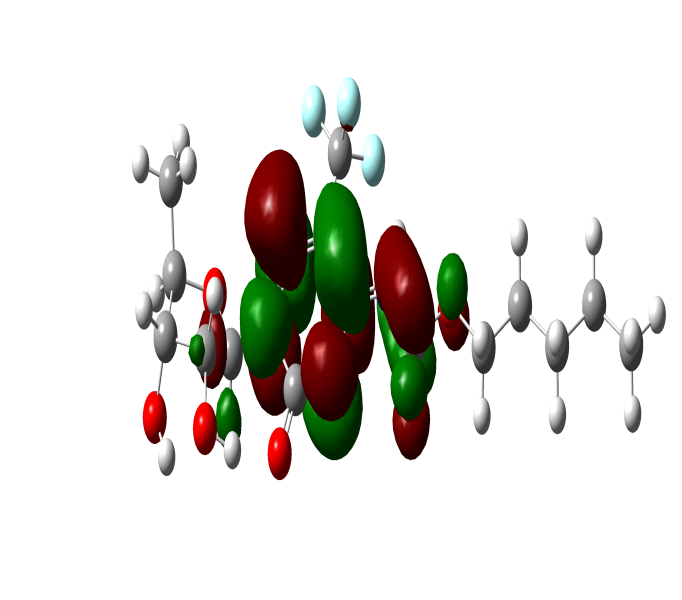 |
| D2 | 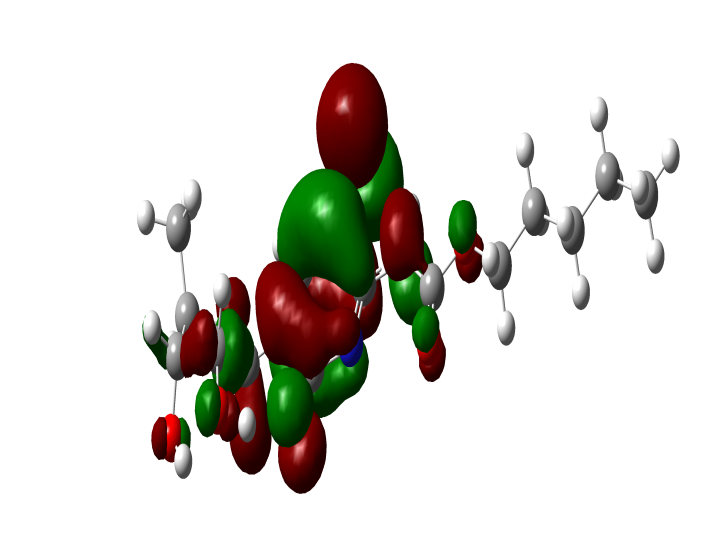 | 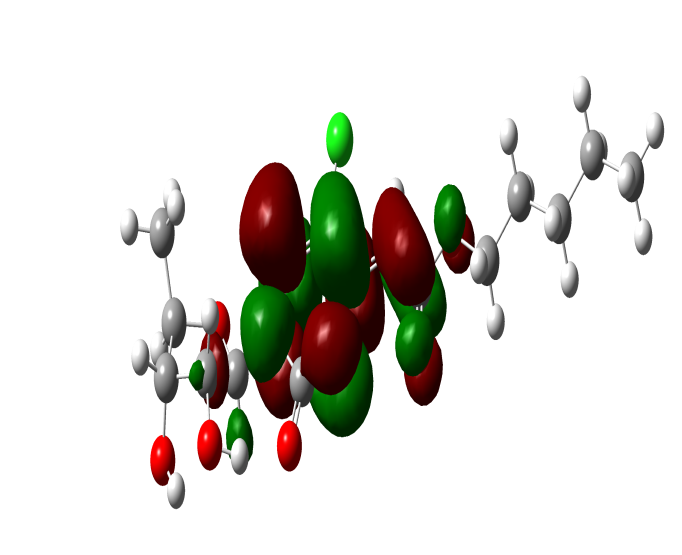 |
| D3 | 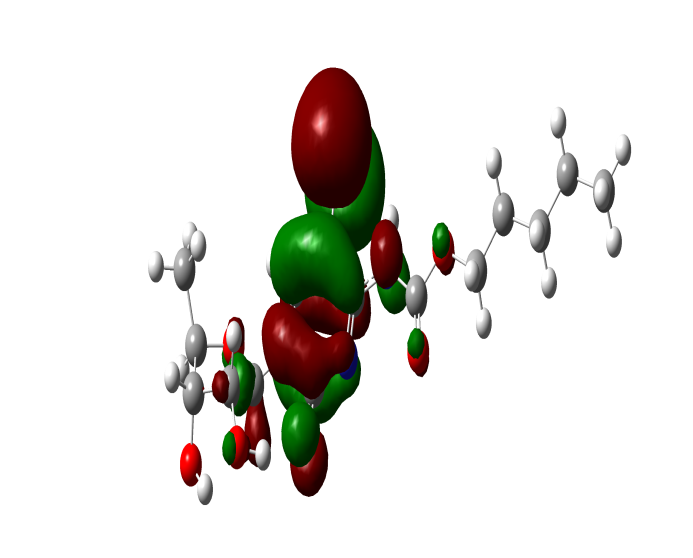 | 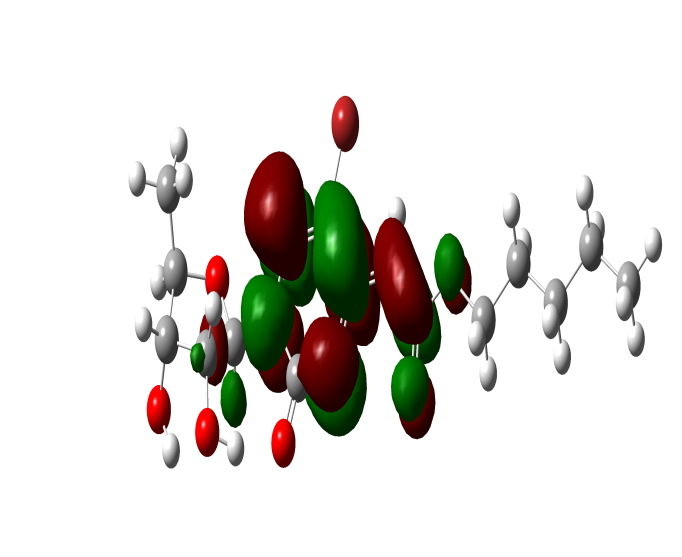 |
| D4 | 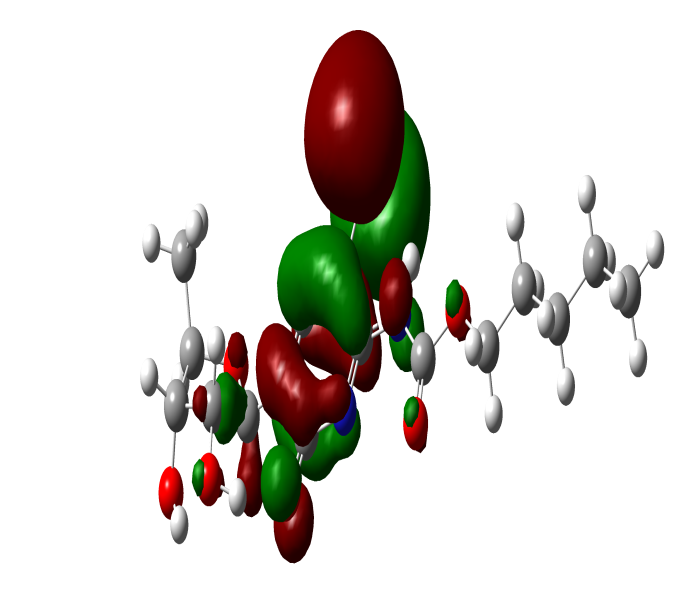 | 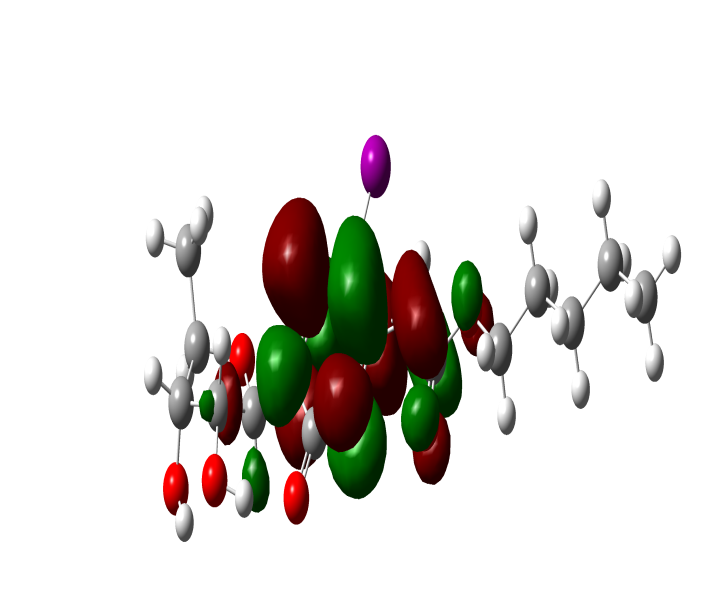 |

1. **D2**-TYMS


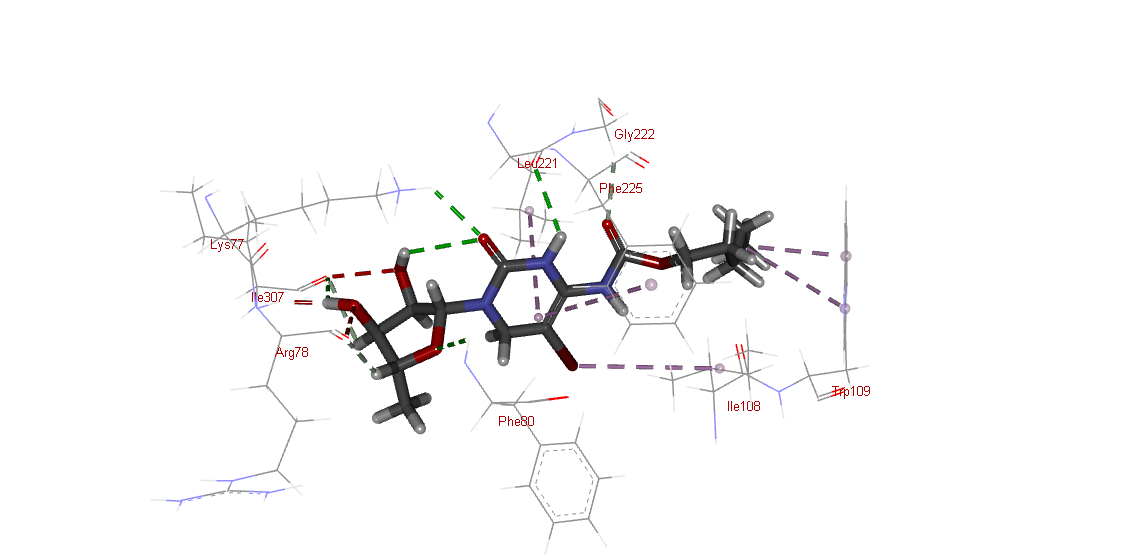


(a) **D3**-TYMS


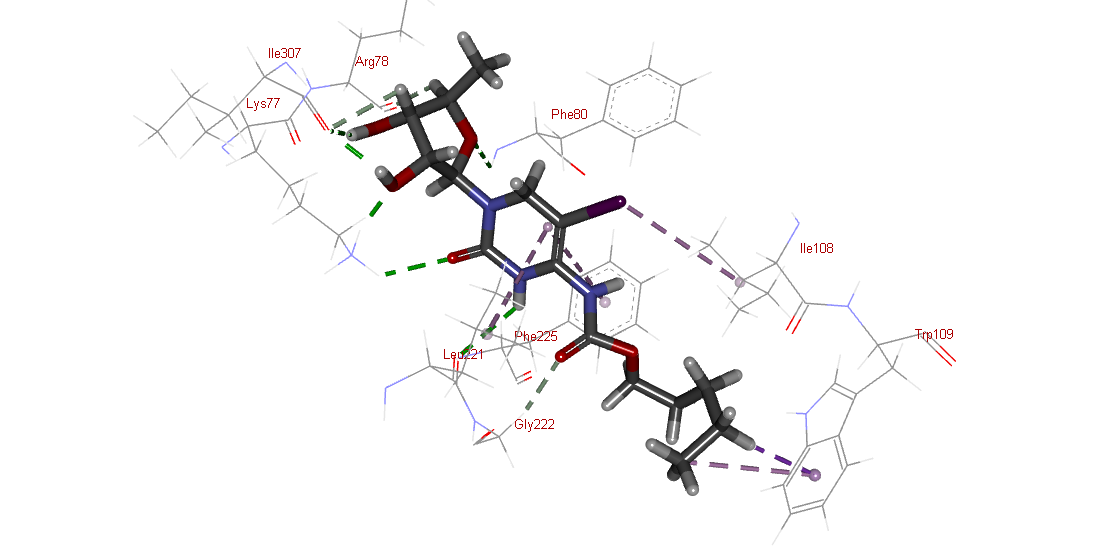


(b) **D4**-TYMS

**Figure S3:** Non-covalent interaction maps of (a) brominated and (b) iodinated capeitabine with TYMS generated by Dicovery Studio

**Figure S4:** Non-bonding Interactions and hydrophobic binding sites of ZD1694-TYMS

( b)

( a)

( c)

( d)

( e)

**Figure S5** : Non-covalent interactions (of rigidly docked ligands) involving (a) Capecitabine, (b) **D1**, (c) **D2**, (d) **D3** and (e) **D4** its with TYMS generated by Ligplot+.
